# Recurrent secondary genomic alterations in desmoplastic small round cell tumors

**DOI:** 10.1186/s12881-020-01034-w

**Published:** 2020-05-11

**Authors:** Warren A. Chow, Jiing-Kuan Yee, Walter Tsark, Xiwei Wu, Hanjun Qin, Min Guan, Jeffrey S. Ross, Siraj M. Ali, Sherri Z. Millis

**Affiliations:** 1 Department of Medical Oncology & Therapeutics Research, City of Hope, 1500 E. Duarte Rd, Duarte, CA 91010 USA; 2 Department of Translational Research & Cellular Therapeutics, City of Hope, Duarte, CA USA; 3 Center for Comparative Medicine, City of Hope, Duarte, CA USA; 4 Integrative Genomics Core of Beckman Research Institute, City of Hope, Duarte, CA USA; 5 Foundation Medicine, Inc, Cambridge, MA USA; 6 Department of Pathology, Upstate Medical University, Syracuse, NY USA

**Keywords:** Desmoplastic small round cell tumor, Sarcoma, Genomic profiling, *FGFR4*

## Abstract

**Background:**

Desmoplastic small round cell tumor (DSRCT) is a rare, highly aggressive, translocation-associated soft-tissue sarcoma that primarily affects children, adolescents, and young adults, with a striking male predominance. It is characterized by t(11;22) generating a novel EWSR1-WT1 fusion gene. Secondary genomic alterations are rarely described.

**Methods:**

Tumor tissue from 83 DSRCT patients was assayed by hybrid-capture based comprehensive genomic profiling, FoundationOne® Heme next generation sequencing analysis of 406 genes and RNA sequencing of 265 genes. Tumor mutation burden was calculated from a minimum of 1.4 Mb sequenced DNA. Microsatellite instability status was determined by a novel algorithm analyzing 114 specific loci.

**Results:**

Comprehensive genomic profiling identified several genomically-defined DSRCT subgroups. Recurrent genomic alterations were most frequently detected in *FGFR4*, *ARID1A*, *TP53*, *MSH3*, and *MLL3* genes. With the exception of *FGFR4*, where the genomic alterations predicted activation, most of the alterations in the remaining genes predicted gene inactivation. No DSRCT were TMB or MSI high.

**Conclusions:**

In summary, recurrent secondary somatic alterations in *FGFR4*, *ARID1A*, *TP53*, *MSH3*, and *MLL3* were detected in 82% of DSRCT, which is significantly greater than previously reported. These alterations may have both prognostic and therapeutic implications.

## Background

Desmoplastic small round cell tumor (DSRCT) is a rare, highly aggressive, translocation-associated soft-tissue sarcoma of children, adolescents, and young adults with a striking ∼85% male bias. Because DSRCT typically presents with diffuse involvement of the abdominal and/or pelvic peritoneum, the 5-year mortality is ~ 85% despite intensive multimodality therapy [1]. The cell of origin remains unknown, but all cases of DSRCT harbor a balanced, reciprocal t(11;22) (p13;q12), resulting in fusion of the N-terminus of the Ewing sarcoma RNA binding protein 1 gene, *EWSR1* (termed *EWS*) to the C-terminus of the Wilms tumor (*WT1*) gene, creating a novel fusion chimera *EWS-WT1* gene [2]. The most common chimera is an in-frame fusion of exons 1–7 of *EWS*, encoding the potential transcription modulating domain, and exons 8–10 of *WT1*, encoding the last three zinc fingers of the DNA-binding domain (reviewed by Loktev et al.) [3].

Despite our understanding of the genomic underpinning of DSRCT, attempts to create a transgenic DSRCT genetically-engineered mouse model (GEMM) using homologous murine *Ewsr1* fused to human *WT1* have been unsuccessful [4]. Similarly, overexpression of EWS-WT1 failed to transform wild-type (wt) primary mouse embryonic fibroblasts (pMEFs), whereas its overexpression in pMEFs with a mutation in at least one allele of transformation related protein 53 (*Trp53*) enhanced proliferation, clonogenic survival, and anchorage-independent growth, consistent with malignant transformation [5]. This suggests that additional unknown somatic aberrations in addition to the *EWS-WT1* chimera gene contribute to oncogenesis. Strikingly however, DSRCTs harbor a low frequency of somatic aberrations [6–9]. For example, Shukla et al. reported 0 somatic mutations in 24 DSRCT tumors analyzed by targeted exon sequencing [6]. Similarly, Jiang et al. reported 2/10 secondary somatic mutations in their DSRCT series (*MET* N375S and *PIK3C* M1040I) using multiple sequencing methods, Silva et al. noted 1/1 *AURKB* and *MCL1* amplification, and Bulbul et al. reported 1/15 (*TP53* G245G) and 1/3 (*FOXO3* L382fs) with a 592-gene next-generation exome sequencing platform [7–9]. This distinctly contrasts with the 32% frequency of *TP53* mutations reported present in other soft-tissue sarcomas [10]. More recent reports using next generation sequencing (NGS) only have reported modestly higher rates of somatic genomic alterations. Using whole exome sequencing (WES), Ferreira et al. noted 1/1 DSRCT with 12 predominantly synonymous and missense somatic mutations [11]. More recently, Devecchi et al. performed WES on 7 DSRCT and reported 8–33 mutations per case [12]. A total of 137 unique somatic mutations were detected, of which 133 were case-specific, and 2 were mutated in two cases but in different positions. Most of the affected genes involved in DNA damage-response network, mesenchymal-epithelial reverse transition (MErT)/epithelial-mesenchymal transition (EMT), and immune response. We describe herein frequent, recurrent, and mostly previously undescribed secondary genomic alterations in DSRCT in the largest clinical database.

## Methods

DSRCT patients whose formalin-fixed and paraffin-embedded (FFPE) tissue was sent for genomic testing between 2012 and 2018 in the course of standard clinical care to Foundation Medicine were included in the analysis. Regardless of prior testing for status of EWS-WT1, only patients whose EWS-WT1 pathognomonic *EWS-WT1* chimera gene status was confirmed during Foundation Medicine testing were included in the cohort. The analysis included both DNA sequencing of 406 cancer-related genes and RNA sequencing of 265 genes commonly rearranged in cancer, as previously described [13]. > 50 ng of DNA and 250 ng of RNA were extracted from the FFPE tissue and assayed by hybrid-capture based next generation sequencing (NGS) analysis on an Illumina HiSeq. Comprehensive genomic profiling (CGP), FoundationOne® Heme, was performed to evaluate for genomic alterations (GAs), including base substitutions, indels, amplifications, copy number alterations and gene fusions/rearrangements. Tumor mutational burden (TMB) was calculated from a minimum of 1.4 Mb sequenced DNA and reported as mutation/Mb. Microsatellite instability status (MSI) was determined by a novel algorithm including 114 specific loci. The clinical status of the patients regarding the source and timing of the specimen acquisition only (primary tumor, metastasis, or recurrence) was provided to Foundation Medicine, however further information regarding the subsequent clinical outcomes were primarily unknown. Approval for this study, including a waiver of informed consent and a HIPAA waiver of authorization, was obtained from the Western Institutional Review Board (Protocol No. 20152817).

RNA sequencing (seq) was performed on a single DSRCT tissue sample under a City of Hope Investigational Review Board approved protocol after written consent was obtained (COH IRB# 15243). The sample was immediately stored in liquid nitrogen after surgery at the COH Tissue Biorepository. This sample was one of the 83 samples sequenced at Foundation Medicine. RNA was isolated using RNeasy MINI kit (Qiagen, Valencia, CA), and RNA-seq was performed at the COH Integrative Genomics Core. RNA-seq libraries were prepared using KAPA Hyperprep RNA-seq kit following manufacturer’s recommendations. The libraries were qualified and loaded to Hiseq 2500 flowcells for single end 51 bp sequencing. The raw sequences were quality filtered and aligned to human genome using Tophat. The expression levels of RefSeq Genes were counted using HTSeq-count. The counts were normalized and differential expression analysis were conducted using Bioconductor package “edgeR”. Pathway analysis and functional annotation of the gene expression data were using GSEA and DAVID, as well as Ingenuity Pathway Analysis.

## Results

**Fig. 1:**
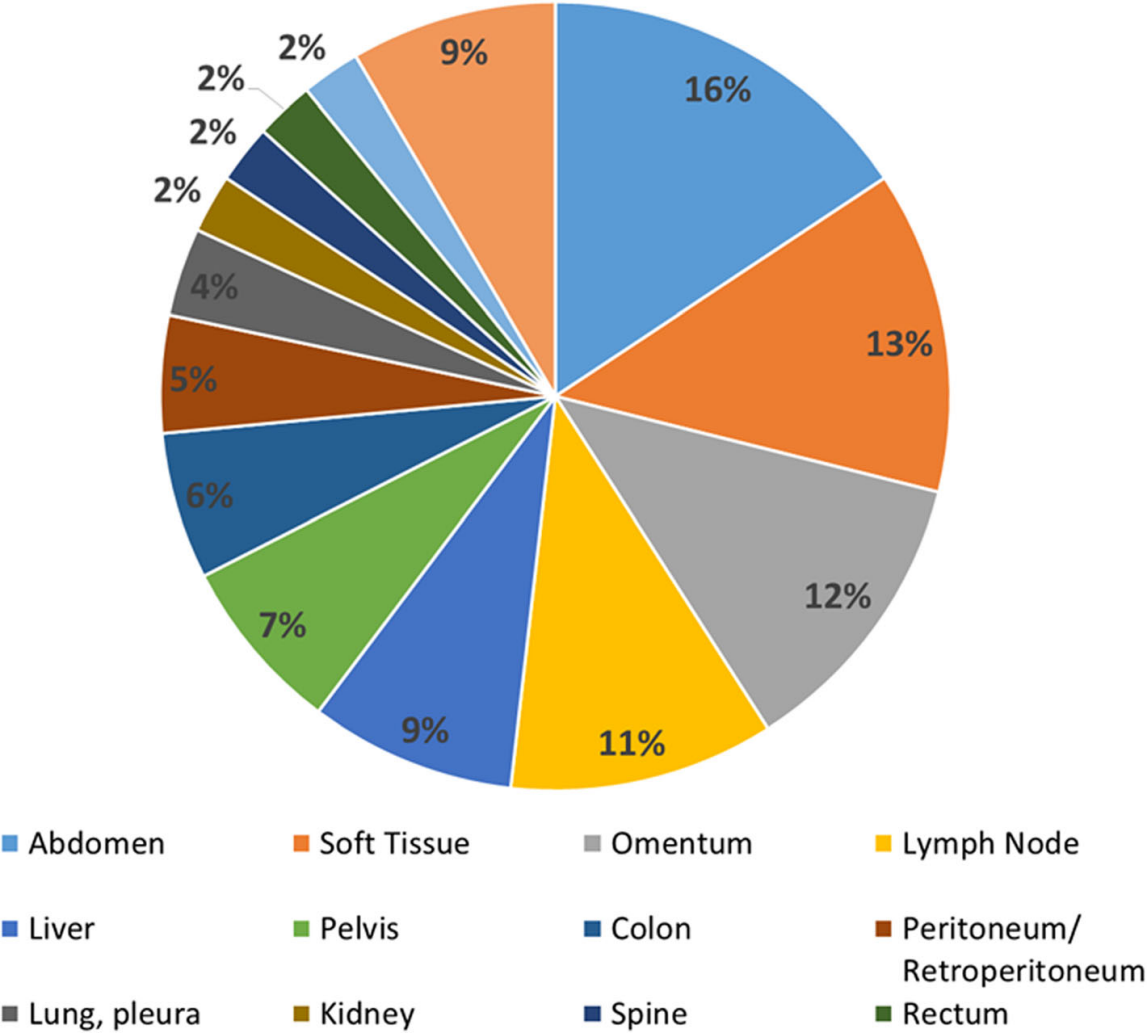
Distribution of specimen sites. Distribution of DSRCT specimen sites sent to Foundation Medicine, Inc. for Comprehensive Genomic Profiling (CGP)

Tissue from 83 DSRCT clinical samples were analyzed at Foundation Medicine. The diagnosis was confirmed by the presence of the *EWS-WT1* chimera gene in all cases by NGS. The demographics of the 83 patient cohort is shown in Table 1. As expected, there was a significant male bias with 81% of the samples from male patients. The median age of the patients was 25 years (range, 6–67). The majority of patients were in the adolescent and young adult (AYA) population (54%). Metastases were clinically documented in 60% of the cases. The distribution of the specimen sites is shown in Fig. 1. The majority of the biopsy samples were taken from the abdomen (16%), soft-tissue (13%), omentum (12%), and lymph nodes (11%). The remainder of the biopsied sites accounted for < 10% each.

**Table 1:** Demographics of 83 patient cohort

	NUMBER	PERCENT
**Gender**		
**Male**	67	81%
**Female**	16	19%
TMB, MSI status*		
**MSS Stable**	All	100%
**TMB Intermediate; > 5, < 20**	5	6%
**TMB Low; < 6**	78	94%
**Median Age (years)**	25	Range 6–67
**Pediatric (< 19)/AYA (19–39)/> 40**	25 / 45 / 13	30% / 54% / 16%
**Metastatic (documented)**	50	60%
**Average number of alterations/patient**	8	Range 1–28

Distribution of age, gender, TMB, microsatellite status, and number of alterations

**Fig. 2:**
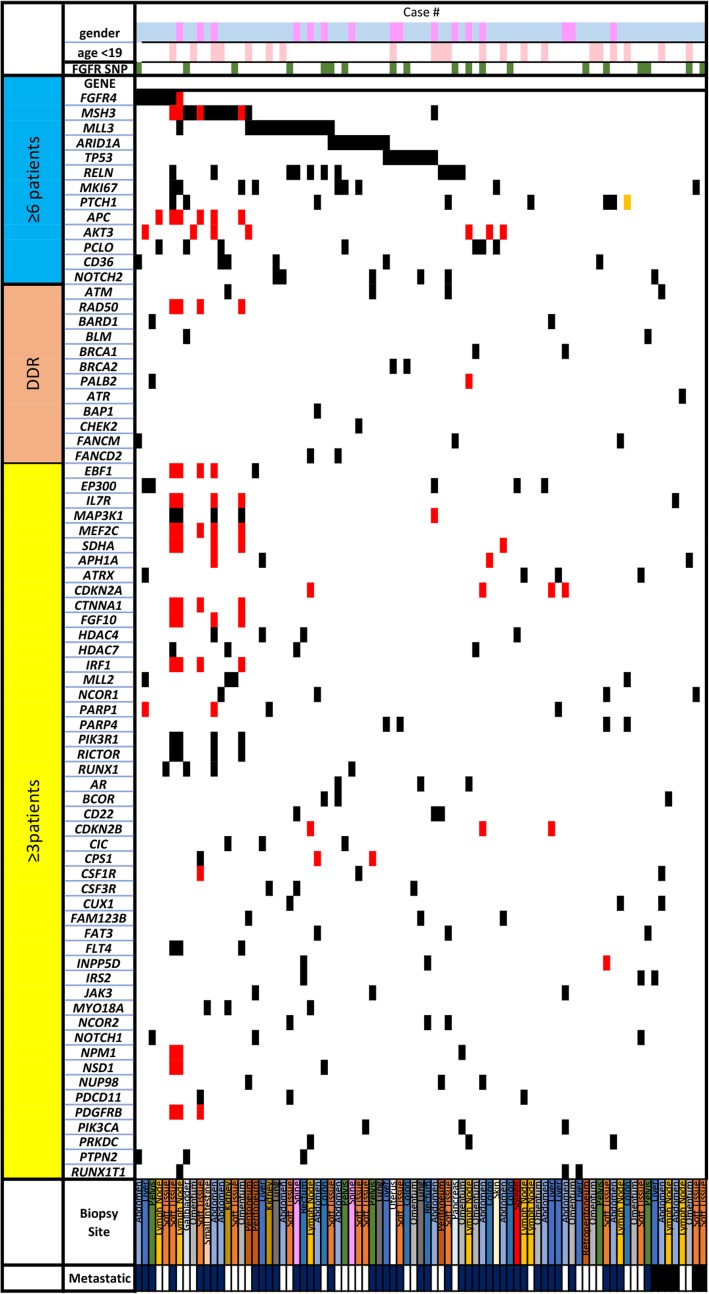
Tile plot. Alterations grouped by: (1) most frequently altered (> 6) with mutual exclusivity to each other (blue color), (2) DNA damage repair (DDR) pathways (salmon color), (3) all other genes (yellow color); to evaluate patterns in distribution and co-incidence. Only genes altered in at least 3 patients are shown. Females, lavender color; age < 19 years, pink color; FGFR4 G385R single-nucleotide polymorphism (SNP), dark green color; single nucleotide variants (SNV), black color; copy number alterations (CN), red color; rearrangements (RE), peach color

CGP identified multiple, recurrent GAs as illustrated in the tile plot (Fig. 2). Only genes altered in at least three patients are shown. Alterations shown are grouped by those with at least 6 patients with mutual exclusivity to each other (blue), DNA damage repair (DDR) pathways (red), and all others (yellow) to evaluate patterns in distribution and co-incidence. The most frequently detected GAs formed several genomically-defined DSRCT subgroups (Table 2). These included: activating mutations, variants of unknown significance (VUS), and amplification of Fibroblast growth factor receptor 4 (*FGFR4*) (*n* = 7; 8%), inactivating mutations of Tumor protein P53 (*TP53*) (*n* = 8; 10%), inactivating alterations of AT-Rich Interaction Domain 1A (*ARID1A*) (*n* = 9; 11%), VUS in MutS Homolog 3 (*MSH3*) (*n* = 12; 14%) and Myeloid/Lymphoid or Mixed-Lineage Leukemia Protein (*MLL3*) (*n* = 13; 16%). The *FGFR4* GAs included: pathogenic activating V510L (*n* = 3; 3.6%), VUS A513V (*n* = 1; 1.2%), VUS N459K (*n* = 1; 1.2%), and amplification (*n* = 2; 2.4%). *ARID1A* alterations and *TP53* alterations were mutually exclusive of *FGFR4* and, with one exception, of each other. While at least 2–3 patients harbored *MLL3* or *MSH3* in addition to either *FGFR4* or *ARID1A*, a majority of those were also mutually exclusive. Genes involved in the DDR pathway formed a separate genomically-defined subgroup, although the number of individual cases for each DDR-related gene were more limited (Fig. 2). The average number of alterations/patients was 8 (range, 1–28). No (0%) DSRCT were TMB High (H, ≥20 mut/Mtb) or MSI High (Table 1).

**Table 2:** Most common genomic alterations

GENE	NUMBER (% of total, ***n*** = 83)	***FGFR4 G388R (% of GA cohort)***
*FGFR4*	*n* = 7 (8%)	*n* = 2 (29%)
*TP53*	*n* = 8 (10%)	*n* = 4 (50%)
*ARID1A*	*n* = 9 (11%)	*n* = 4 (44%)
*MSH3*	*n* = 12 (14%)	*n* = 6 (50%)
*MLL3*	*n* = 13 (16%)	*n* = 6 (46%)
Other GA	*n* = 34	*n* = 19 (56%)

Frequency of the most common, recurrent GAs detected in DSRCT (group 1) that have been reported to contribute to oncogenesis in other cancers

RNA-seq performed on a single patient at City of Hope, as part of institutional-approved clinical research, identified a G-to-A single nucleotide polymorphism (SNP) at codon 388 (Gly to Arg) [*FGFR4* G388R], which was confirmed by Sanger sequencing. This SNP has been implicated in the progression and prognosis of multiple human cancers, including soft-tissue sarcomas [14–18]. It is considered oncogenic, as pMEFs from homologous *FGFR4Arg385* knock-in mice are transformed [19]. This SNP is not normally reported by the FoundationOne Heme® platform, as it is present in the normal population at an allele frequency of 32.1% in the Exome Aggregation Consortium (http://exac.broadinstitute.org/variant/5-176520243-G-A). Re-query of the platform with this information led to the identification of 19/34 (56%) additional tumors without other secondary genomic alterations from the group with mutual exclusivity (blue) with the *FGFR4* G388R SNP detected (Table 2).

## Discussion

In the largest DSRCT series using CGP to date, multiple, recurrent secondary GAs were identified in the majority of clinical samples. The most frequently identified GAs can be broadly classified as: (1) potential oncogenes (i.e., *FGFR4*); (2) tumor suppressor genes (i.e., *TP53* and *ARID1A*); (3) GAs of unknown clinical significance (*MSH3* and *MLL3*). Less frequently identified GAs were classified as (4) DDR pathway genes and (5) all others. With the exception of *TP53* and *ARID1A*, the remainder of the GAs have not previously been noted [5–8, 12], likely due to limited gene sequencing. The results suggest the possibility of heretofore unidentified actionable mutations that may have significant implications upon DSRCT oncogenesis and the discovery of potential therapeutic targets.

*FGFR4* is a member of the *FGFR* family (*FGFR1, FGFR2, FGFR3* and *FGFR4*); however, the tyrosine kinase domain structurally different enough from *FGFR1–3* that small molecule FGFR1–3 inhibitors are generally incapable of suppressing FGFR4 activation at similarly effective nanomolar concentrations [20]. Relatively frequent mutations in *FGFR2* (10% of endometrial cancer) and *FGFR3* (20% of urothelial cancer), gene fusion in *FGFR2* (45% of intrahepatic cholangiocarcioma) and gene amplification in *FGFR1* (19% of ER-positive breast and 17% of squamous cell lung cancer) and *FGFR2* (< 10% of gastric cancer) have been described [20]. GAs in *FGFR4* have been described in 6.1% of rhabdomyosarcomas (RMS) overall, but is enriched (9.6% in the *PAX* gene fusion negative subset (generally embryonal subtype) [21]. In a separate analysis, 7.5% of primary RMS contained a tyrosine kinase domain mutation, and the particular mutants K535 and E550 increased tumor proliferation, metastatic potential, autophosphorylation, and Stat3 signaling when expressed in a murine RMS cell line [22]. These results confirm the oncogenic potential of activated FGFR4.

The *FGFR4* G388R SNP was originally discovered by Bange et al. and colleagues to be associated with tumor progression in breast and colon cancer patients [23]. Subsequently, this SNP has been reported to be associated with advanced stage and poor prognosis in patients with carcinomas of the lung, prostate, and head and neck; melanomas; and soft-tissue sarcomas [14–18]. This association was confirmed in two large meta-analyses and a pooled analysis of 2537 cancer cases [24, 25]. A causative relationship for this SNP to cancer progression was demonstrated when pMEFs from homologous *FGFR4Arg385* knock-in mice were shown by Seitzer et al. to accelerate cell transformation with greater motility and invasive behavior [19]. In vivo, transforming growth factor (TGF)α-induced mammary carcinogenesis, tumor development and progression, and onset of pulmonary metastases were significantly advanced [19]. Later, Ulaganathan et al. established the underlying pathobiology of the SNP; substitution of the conserved human Gly 388 residue to a charged Arg residue modified the transmembrane spanning segment and exposed a membrane-proximal cytoplasmic signal transducer and activator of transcription 3 (STAT3) binding site Y^390^-(P)XXQ^393^ [26]. Such STAT3 binding motifs in the germline of type 1 membrane receptors enhance STAT3 activation by recruiting STAT3 proteins to the inner cell membrane. Enhanced STAT3 signaling induced by *FGFR4* G388R was confirmed in vivo with the *FGFR4Arg385* knock-in mice and transgenic mouse models for breast and lung cancers [26]. These results confirm the oncogenic potential of *FGFR4* G388R. Interestingly, when the current DSRCT series was queried for the frequency of this SNP within each genomic alteration cohort, its frequency, albeit with a small sample size, approximated the normal population (32.1%) in the *FGFR4* cohort (29%), but was overrepresented within all the other cohorts (44–50%) (Table 2). The significance of this finding suggests the hypothesis that *FGFR4* genomic alterations (activating mutations, amplification, or SNP) are sufficient as the “second hit” in translocation-positive cells, whereas there may be an additional requirement for a “third hit” with the *FGFR4* SNP in up to half of the other genomic alterations.

*TP53* is a tumor suppressor gene, and its inactivation is a frequent event in tumorigenesis [27]. We detected inactivating mutations in *TP53* at greater frequency in DSRCT (10%) than previously reported. Jiang et al. reported 0/10 DSRCT samples with *TP53* mutations, whereas Bulbul et al. reported 1/15 (7%) DSRCT samples with *TP53* mutations [7, 9]. The greater frequency in the current series may simply be related to the larger sample size. Nevertheless, both the present frequency and the other reported frequencies are significantly lower than reported for other soft-tissue sarcomas (32%) [10]. The biology underlying the low frequency of *TP53* mutations in DSCRCT compared to other soft-tissue sarcomas is unclear.

ARID1A is one of two mutually exclusive ARID1 subunits of the adenosine triphosphate-dependent chromatin modeling complex switch/sucrose-nonfermentable (SWI/SNF), which acts to mobilize nucleosomes and regulates gene expression and chromatin dynamics [28]. ARID1A is thought to provide specificity to this complex [28]. *ARID1A* mutations were originally described at high frequency in ovarian clear cell carcinoma (OCCC), an uncommon but aggressive subtype of ovarian cancer. Subsequently, genomic alterations in *ARID1A* have been described in a broad array of tumor types with the notable exception of sarcomas [29]. ARID1A participates in directing at least 3 processes relevant to tumor suppression: proliferation, differentiation, and apoptosis [28]. Accordingly, it has been labeled an epigenetic tumor suppressor [28]. A single *ARID1A* nonsense mutation was detected in 1/7 DSRCTs by Devecchi et al. [12], whereas in the current series *ARID1A* inactivating mutations (truncation or indels) were the third most frequent GA detected in 11% of DSRCT samples underscoring the significance of a larger sample size.

VUS in *MSH3* and *MLL3* were detected in 14 and 16% of DSRCT samples respectively, accounting for the most frequent genomically-defined subgroups. MSH3 forms a heterodimer with MSH2 to form MutS-β, which comprises part of the post-replicative DNA mismatch repair system. Inactivating mutations of *MSH3* is considered a low-risk allele that contributes to development of hereditary nonpolyposis colorectal cancer (HNPCC), or Lynch syndrome [30]. Patients with HNPCC have an increased lifetime risk of developing colorectal cancer, as well as cancers of the endometrium, liver and biliary tract, stomach, small intestine, ovary, ureters, renal pelvis, and brain [30]. *MLL3* is a member of the myeloid/lymphoid or mixed-lineage leukemia (*MLL*) family comprising a nuclear protein with an AT hook DNA-binding domain, a SET domain, a post-SET domain, a DHHC-type zinc finger, six PHD-type zinc fingers, and a RING-type zinc finger [31]. It is a member of the ASC-2/NCOA6 complex (ASCOM), and is involved in transcriptional co-activation through regulation of histone methylation [31]. *MLL3* was recently shown to act as a haploinsufficient tumor suppressor gene in − 7/del(7q) acute myeloid leukemia [31]. As recurrent mutations in *MSH3* and *MLL3* have not been described for sarcomas, their exact role in the pathobiology of DSRCT remains unclear. Regardless, given their roles in other cancers, we suspect the GAs detected are inactivating in DSRCT.

Genes associated with the DDR pathway formed a fourth subgroup of GAs. However, the number of individual cases for each DDR-related gene were limited. Devecchi et al. reported 26 unique somatic mutations in genes involved in the DDR network in 6 of 7 DSRCT cases, including one each of *ATR*, *TP53*, and *ARID1A* [12]. GAs in these genes were also detected in the current CGP, however the remainder of the DDR genes reported here are unique. It is unclear whether the current DDR GAs are driver mutations, passenger mutations, or a result of therapy-induced alterations. Further research into the significance of these DDR genes in DSRCT oncogenesis is necessary.

The role of immunotherapy for sarcomas remains investigational. To date, the limited efficacy of anti-PD1 blockade in other soft-tissue and bone sarcomas has not been reported for DSRCT [32]. In the course of standard clinical care for DSRCT, genomic analysis including TMB and MSI analysis were performed as part of the FoundationOne® Heme panel as TMB High and MSI High have been positively correlated with response to anti-PD1 blockade therapy in other cancers [32]. The results demonstrated the tumors were neither TMB High nor MSI High. These results are consistent with recent reports that DSRCT had low TMB consistent with low immunogenicity, and carry a miRNA signature of immunological ignorance that is not responsive to PD-L1 blockade [9, 33].

The ongoing status of the patients regarding their clinical course were not provided to Foundation Medicine; therefore correlation of the identified genomic alterations to patient outcomes were not available. Certainly, it would be of significant interest should any of these genomic alterations have prognostic value. However, given the limited clinical information in the current series, it is impossible to evaluate. Specifically designed retrospective studies or future prospective studies will be able to determine the clinical significance of these findings.

## Conclusions

In sum, recurrent secondary GAs alone, *FGFR4* G388R SNP alone, or the combination of secondary GA and *FGFR4* G388R SNP were identified in the vast majority of DSRCT samples (*n* = 68; 82%) when more comprehensive genomic profiling was performed. The most frequently observed GAs formed several genomically-defined subgroups, all of which have been reported to contribute to oncogenesis in other cancers. Their precise role in DSRCT oncogenesis is currently under active laboratory investigation, and their prognostic and predictive values should be investigated in future clinical studies.

## Data Availability

The datasets generated and analyzed during the current study are not publicly available due to HIPAA regulations, but are available from the corresponding author on reasonable request and approval of Foundation Medicine, Inc., Cambridge, MA, U.S.A.

## Abbreviations

*ARID1A*: AT-rich interaction domain 1A gene
ASCOM: ASC-2/NCOA6 complex
*AURKB*: Aurora B kinase gene
AYA: Adolescent and young adult
CGP: Comprehensive genomic profiling
DDR: DNA damage repair
DSRCT: Desmoplastic small round cell tumor
EMT: Epithelial-mesenchymal transition
*EWS*: Ewing sarcoma RNA binding protein 1 gene
FFPE: Formalin-fixed and paraffin-embedded
*FGFR4*: Fibroblast growth factor receptor 4 gene
*FOXO3*: Forkhead box 03 gene
GAs: Genomic alterations
GEMM: Genetically-engineered mouse model
HIPAA: Health Information Portability and Accountability Act
HNPCC: Hereditary nonpolyposis color rectal cancer
*MCL1*: Induced myeloid leukemia cell differentiation gene
MErT: Mesenchymal-epithelial reverse transition
*MET*: Hepatocyte growth factor receptor gene
*MLL3*: Myeloid/lymphoid or mixed-lineage leukemia protein gene
*MSH3*: MutS Homolog 3 gene
MSI: Microsatellite instability
NGS: Next generation sequencing
*PAX*: Paired box gene
*PIK3C*: Phosphatidylinositol-4,5-bisphosphate 3-kinase, catalytic subunit alpha gene
pMEFs: Primary mouse embryonic fibroblasts
RMS: Rhabdomyosarcoma
RNA-seq: RNA sequencing
SNP: Single nucleotide polymorphism
*Stat3*: Signal transduced and activator of transcription 3 gene (murine)
*STAT3*: Signal transduced and activator of transcription 3 gene
SWI/SNF: Switch/sucrose-nonfermentable gene
*TMBTP53*: Tumor protein 53
TGFα: Transforming growth factor α
*Trp53*: Transformation related protein 53 gene (murine)
VUS: Variants of unknown significance
WES: Whole exome sequencing
wt: Wild-type
*WT1*: Wilm’s Tumor gene
